# Biological Data Resources and Machine Learning Frameworks for Hematology Research

**DOI:** 10.1093/gpbjnl/qzaf021

**Published:** 2025-03-04

**Authors:** Ying Yi, Yongfei Hu, Juanjuan Kang, Qifa Liu, Yan Huang, Dong Wang

**Affiliations:** Institute of Dermatology and Venereology, Dermatology Hospital, Southern Medical University, Guangzhou 510091, China; Institute of Dermatology and Venereology, Dermatology Hospital, Southern Medical University, Guangzhou 510091, China; Department of Bioinformatics, Guangdong Province Key Laboratory of Molecular Tumor Pathology, School of Basic Medical Sciences, Southern Medical University, Guangzhou 510515, China; Department of Hematology, Nanfang Hospital, Southern Medical University, Guangzhou 510515, China; Cancer Research Institute, School of Basic Medical Sciences, Southern Medical University, Guangzhou 510515, China; Institute of Dermatology and Venereology, Dermatology Hospital, Southern Medical University, Guangzhou 510091, China; Department of Bioinformatics, Guangdong Province Key Laboratory of Molecular Tumor Pathology, School of Basic Medical Sciences, Southern Medical University, Guangzhou 510515, China

**Keywords:** Hematopoiesis, Hematologic disorder, Biological resource, Clinical resource, Machine learning

## Abstract

Hematology research has greatly benefited from the integration of diverse biological data resources and advanced machine learning (ML) frameworks. This integration has not only deepened our understanding of blood diseases such as leukemia and lymphoma, but also enhanced diagnostic accuracy and personalized treatment strategies. By applying ML algorithms to analyze large-scale biological data, researchers can more effectively identify disease patterns, predict treatment responses, and provide new perspectives for the diagnosis and treatment of hematologic disorders. Here, we provide an overview of the current landscape of biological data resources and the application of ML frameworks pertinent to hematology research.

## Graphical abstract

**Figure:**
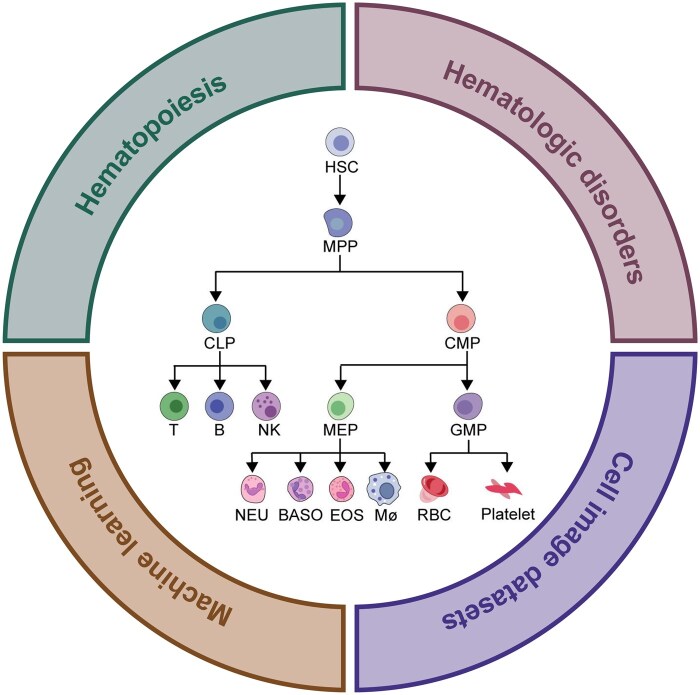

## Introduction

Hematology is a subject area for the study of blood and its related diseases. It includes, but is not limited to, the following aspects: (1) understanding the physiological functions of the blood system, including blood composition, blood circulation, and coagulation mechanism [1,2]; (2) revealing the pathogenesis of hematologic diseases [3–5]; (3) promoting the research and development of new drugs and therapeutic methods [6]; and (4) exploring the use of blood markers for the diagnosis of diseases and prognostic assessment [7]. The onset, development, diagnosis, treatment, and regression of many diseases are closely related to the blood system, and many biomedical technologies begin with hematology research [8].

The development of high-throughput technologies in multi-omics fields (genomics, transcriptomics, proteomics, and epigenomics) has led to the exponential growth of biological data and the continuous expansion of existing resources [9]. Advances in science and technology have also made it easier to integrate and link different types and sources of biological data, resulting in a more comprehensive and integrated biomedical data resource and improved data utilization. Comprehensive public databases such as The Cancer Genome Atlas (TCGA) [10] contain a large amount of data on cancer samples, including multi-omics sequencing data as well as clinical information. The Human Protein Atlas [11] provides information on the tissue and cellular localization of human protein expression. Single Cell Atlas (SCA) [12] is an open-access single-cell multi-omics healthy human atlas. RNA Interactome Database (RNAInter) 4.0 [13] is an RNA interactome repository covering 156 species. Online Mendelian Inheritance in Man (OMIM) [14] and ClinVar [15] are human disease-related databases, and Orphanet database [16] is a knowledge base on rare diseases and orphan drugs. Notably, the growth of digital pathology and imaging technologies has led to an abundance of blood cell image datasets, enabling researchers to develop sophisticated image analysis techniques [17–19].

**Figure 1:**
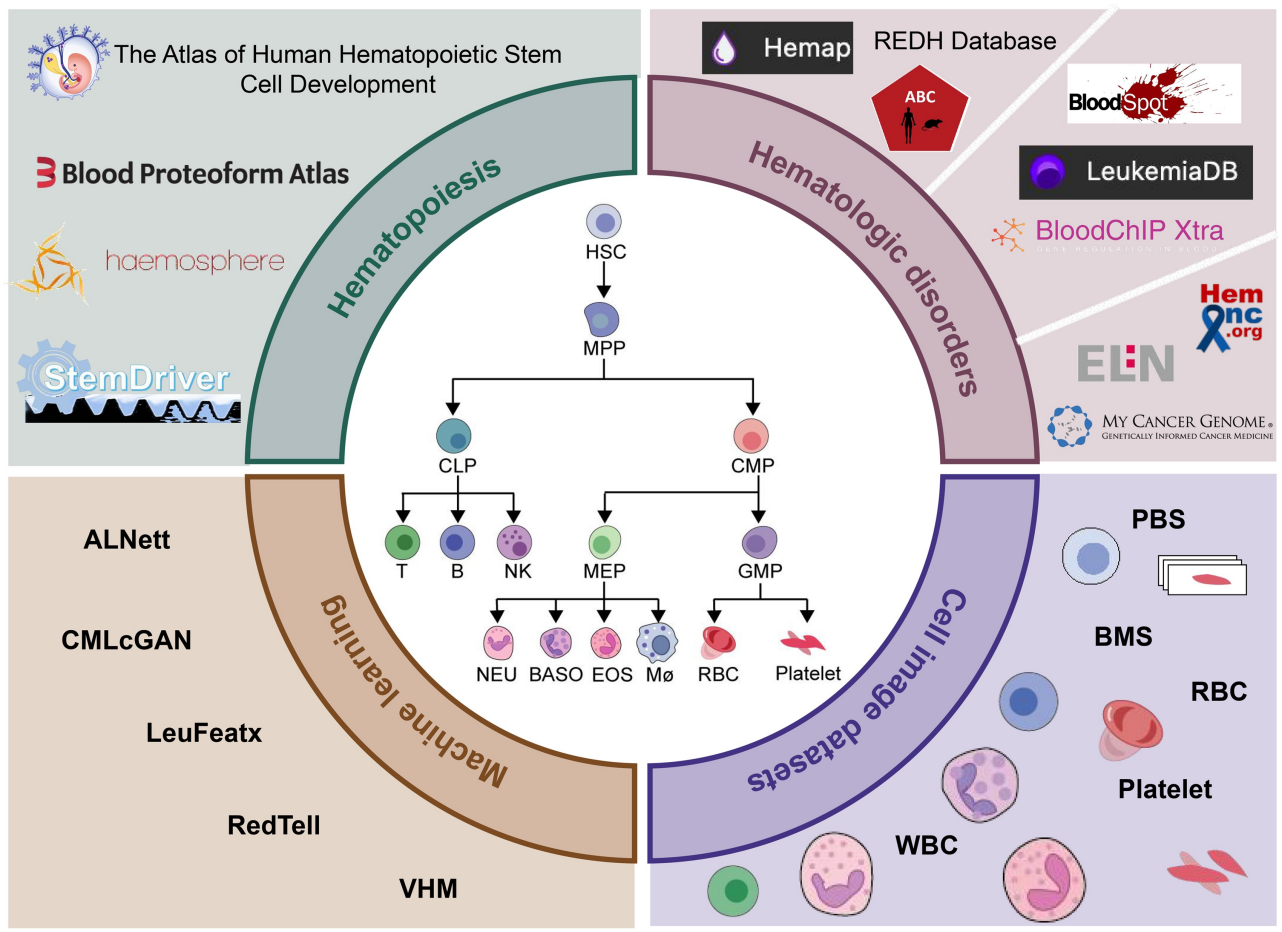
The overview of data resources/datasets and machine learning frameworks in this review PBS, peripheral blood smear; BMS, bone marrow smear; WBC, white blood cell; RBC, red blood cell; HSC, hematopoietic stem cell; MPP, multipotent progenitor cell; CLP, common lymphoid progenitor; CMP, common myeloid progenitor; NK, natural killer cell; MEP, megakaryocyte–erythrocyte progenitor; GMP, granulocyte–macrophage progenitor; NEU, neutrophil; BASO, basophil; EOS, eosinophil; MΦ, macrophage; ELN, European LeukemiaNet; REDH, RNA Editome in Hematopoietic differentiation and malignancy; LeuFeatx, Leukocyte Feature-Extraction model; ABC, Atlas of Blood Cells; VHM, virtual hematological morphologist.

Machine learning (ML) plays a pivotal role in interpreting these complex datasets, offering innovative ways to identify patterns and predict patient outcomes. In particular, ML is being increasingly integrated with blood cell image datasets to enhance diagnostic precision, with deep learning models showing great promise in recognizing cell morphologies indicative of disease [20,21]. Furthermore, ML frameworks have proven instrumental in analyzing omics data (such as genomic, transcriptomic, and proteomic profiles) to identify molecular biomarkers and therapeutic targets for hematologic disorders. The integration of clinical data with ML models allows for more accurate patient stratification and personalized treatment regimens [22]. Here, we highlight key data resources in hematology, focusing on hematopoiesis, hematologic malignancies, leukemia-specific data, comprehensive clinical resources, and blood cell image datasets (**Figure 1**; **Tables 1** and **2**). It explores how the integration of ML with biological and clinical data paves the way for more accurate diagnostics, improved treatment outcomes, and personalized medicine in the field of hematology.

**Table 1:** Summary of biological databases for hematology research

Name	URL	Data source	Data content	Key feature	Limitation	Citation
Atlas of Human Hematopoietic Stem Cell Development [23]	https://singlecell.mcdb.ucla.edu/Human-HSC-Ontogeny/	scRNA-seq and spatial transcriptomics	A single-cell transcriptome map of human hematopoietic tissues from the first trimester to birth	Gene expression during development of HSCs from HE to birth	Limited to developmental stages	144
Haemosphere [24,25]	https://www.haemosphere.org	Microarray and RNA-seq	1 RNA-seq gene expression dataset (129 samples) and 1 microarray dataset (169 samples) covering progenitors and all the major mature hematopoietic lineages in mice; 1 RNA-seq gene expression dataset (42 samples) covering 7 mature lineages in humans	Gene expression datasets from diverse hematopoietic cells in mice and humans	Focus only on transcriptomics	113, 138
BPA [26]	https://blood-proteoform-atlas.org/	Proteomics	29,620 non-redundant proteoforms and 1690 proteins across 21 different human hematopoietic cell types and plasma components	A reference map of proteoforms in human hematopoietic cells	Samples are primarily from healthy donors, lacking disease representation	90
StemDriver [27]	http://biomedbdc.wchscu.cn/StemDriver/	scRNA-seq	42 scRNA-seq datasets from human and mouse samples spanning 22 major cell types in hematopoiesis	Comprehensive gene annotation focusing on HSC fate determination in humans and mice	Limited cell type coverage and lack of experimental validation	1
Hemap [28]	http://hemap.uta.fi/	scRNA-seq	7279 transcriptomes from 36 hematologic malignancies (leukemia, lymphoma, myeloma, and proliferative disorders), 862 from cancer cell lines and 1403 from normal blood cell types	Analyzing gene expression, pathway activity, and drug response characteristics in hematologic malignancies	Primarily rely on public data	30
ABC portal [29]	http://abc.sklehabc.com	scRNA-seq	111 datasets of hematopoiesis and development, 87 datasets of blood disease	A specialized single-cell database for human and mouse blood cells, featuring unified cell type annotations and customizable reanalysis tools	Restricted to single-cell transcriptomics	16
BloodSpot 3.0 [34]	https://www.fobinf.com/	Microarray, RNA-seq, scRNA-seq, and single-cell proteomics	Gene and protein expression datasets in normal and malignant hematopoiesis	Visualization of gene and protein expression in healthy hematopoiesis and leukemia, including pediatric leukemias	Lack of direct access to datasets	8
BloodChIP Xtra [35]	http://bloodchipxtra.vafaeelab.com/	ChIP-seq, ATAC-seq, and RNA-seq	4 ATAC-seq datasets, 9 ChIP-seq datasets, and 4 RNA-seq datasets in rare primary human HSCs (HSC-MPP), progenitors (CMP, GMP, MEP), and AML cell lines	Exploration and visualization of TF occupancy, chromatin accessibility, and gene expression data across HSC-MPP, CMP, GMP, and MEP cells, as well as AML cell lines	Inadequate coverage for other hematopoietic disorders	3
LeukemiaDB [37]	http://bioinfo.life.hust.edu.cn/LeukemiaDB	RNA-seq	188 leukemia-associated RNA-seq datasets covering 14 leukemia subtypes and 53 leukemia cell lines	A comprehensive leukemia transcriptome data resource, including expression profiles for protein-coding genes, lncRNAs, and cirRNAs, as well as fusion and AS events	Lacking more advanced analytical tools, limiting the flexibility for high-level analyses	3
REDH Database [30]	http://www.redhdatabase.com	RNA-seq	30,796 RNA editing sites among 12 murine adult hematopoietic cell populations and 435,866 aberrant editing sites associated with leukemia (20 AML samples, 9 CML samples, and 19 healthy donors)	RNA editing events in mouse hematopoiesis and human leukemia	Limited types of hematopoietic malignancies	0
HemOnc.org [40]	https://hemonc.org/wiki/Main_Page	Clinical data	5351 treatment regimens and 1217 CPGs	Clinical guidelines and chemotherapy protocols for hematology and oncology	Limited to textual guidelines	49
ELN [41]	https://www.leukemia-net.org/	Clinical data	Over 100 management recommendations and guidelines developed by more than 1000 physicians and researchers and 220 institutions	European Leukemia Network guidelines and resources	Focus on European data and protocols	32
MCG [42]	https://www.mycancergenome.org/	Clinical data	957 disease types (including 220 hematopoietic and lymphatic system neoplasms), 3324 biomarkers, 9852 clinical trials, 2876 drugs, and 19 biological pathways	A precision cancer medicine knowledge resource, including diseases, biomarkers, drugs, pathways, and clinical trials	Limited pediatric data	23

*Note*: scRNA-seq, single-cell RNA sequencing; RNA-seq, RNA sequencing; HSC, hematopoietic stem cell; HE, hemogenic endothelium; BPA, Blood Proteoform Atlas; ABC, Atlas of Blood Cells; ChIP-seq, chromatin immunoprecipitation sequencing; ATAC-seq, assay for transposase-accessible chromatin with high-throughput sequencing; MPP, multipotent progenitor cell; CMP, common myeloid progenitor; GMP, granulocyte–macrophage progenitor; MEP, megakaryocyte–erythrocyte progenitor; AML, acute myeloid leukemia; TF, transcription factor; lncRNA, long non-coding RNA; circRNA, circular RNA; AS, alternative splicing; CML, chronic myelogenous leukemia; CPG, clinical practice guideline; MCG, My Cancer Genome; ELN, European LeukemiaNet; REDH, RNA Editome in Hematopoietic differentiation and malignancy.

**Table 2:** List of the publicly available blood cell image datasets

Dataset	Application	No. of images	Image format	Access
ALL-IDB dataset [43]	Cell segmentation Cell classification	ALL-IDB1: 108 PBS images ALL-IDB2: 260 acute lymphoblast images	JPG	https://scotti.di.unimi.it/all/
BCCD Dataset	Cell detection	360 labeled images for WBCs, RBCs, and platelets	JPEG	https://github.com/Shenggan/BCCD_Dataset
Chula-RBC-12-Dataset [44]	Cell detection and classification	738 PBS images containing 20,875 RBCs	JPG	https://github.com/Chula-PIC-Lab/Chula-RBC-12-Dataset
Raabin-WBC [45]	Cell classification	40,764 WBC images (double-labeled Raabin-WBC: 17,965 WBC images)	JPG	https://www.raabindata.com/free-data/
	Cytoplasm and nucleus segmentation	1145 cell images	JPG, BMP	
PBS_HCB dataset [46]	Cell classification	17,092 normal cell images from neutrophils, eosinophils, basophils, lymphocytes, monocytes, immature granulocytes, erythroblasts, and platelets	JPG	https://data.mendeley.com/datasets/snkd93bnjr/1
BMCD-FGCD dataset [47]	Cell classification	92,335 bone marrow blood cell images spanning nearly 40 distinct blood cell categories	PNG	https://drive.google.com/file/d/1hOmQ9s8eE__nqIe3lpwGYoydR4_UNRrU/view?usp=drive_link
Microscopic Images of Multiple Myeloma [48]	Cell segmentation	85 BMS images of multiple myeloma	BMP	https://www.cancerimagingarchive.net/collection/mimm_sbilab/
Blood Cells Cancer (ALL) dataset [49]	Cell classification	3242 PBS images from 89 patients suspected of ALL	JPG	https://www.kaggle.com/datasets/mohammadamireshraghi/blood-cell-cancer-all-4class
RBC dataset [50]	Cell classification	7108 RBC images from 4 thalassemia PBSs and a healthy PBS	PNG	https://data.mendeley.com/datasets/rfdz6wfzn4/1
AML-Cytomorphology dataset [51]	Cell classification	18,365 cell images from PBSs of 100 AML patients as well as 100 patients without signs of hematological malignancy	TIFF	https://www.cancerimagingarchive.net/collection/aml-cytomorphology_lmu/
100K-RBC-Mask-PathOlOgics dataset [52]	Cell segmentation	100,118 RBC images from patients suspected to have PMF	JPG	https://doi.org/10.6084/m9.figshare.24119511.v1
100K-RBC-PathOlOgics dataset [52]	Cell classification	100,873 RBC images from patients suspected to have PMF	JPG	https://doi.org/10.6084/m9.figshare.24119511.v1
Bone-Marrow-Cytomorphology dataset [53]	Cell classification	171,375 cell images from the BMSs of 945 patients	JPG	https://www.cancerimagingarchive.net/collection/bone-marrow-cytomorphology_mll_helmholtz_fraunhofer/

*Note*: ALL-IDB, acute lymphoblastic leukemia image database for image processing; BCCD, blood cell count and detection; PBS_HCB, microscopic peripheral blood cell image dataset from the Hospital Clinic of Barcelona; BMCD-FGCD, bone marrow blood cell fine-grained classification dataset; WBC, white blood cell; RBC, red blood cell; PBS, peripheral blood smear; BMS, bone marrow smear; ALL, acute lymphoblastic leukemia; AML, acute myeloid leukemia; PMF, primary myelofibrosis.

## Data resources on hematology research

### Resources on hematopoiesis

#### Atlas of Human Hematopoietic Stem Cell Development

The Atlas of Human Hematopoietic Stem Cell (HSC) Development is a single-cell transcriptome map of human hematopoiesis created by Calvanese et al. [23], covering the development, migration, and maturation of human HSCs from hemogenic endothelium to the hematopoietic microenvironment. Expression data are available at the Gene Expression Omnibus (GEO) under accessions GSE162950 and GSE135202. They also built an online interface (http://singlecell.mcdb.ucla.edu/Human-HSC-Ontogeny) to provide visualization of the research findings of this study. Through this atlas, users can gain a deeper understanding of the origin and development of HSCs and explore the molecular mechanisms of certain blood disorders. For example, it can help identify gene markers that distinguish HSCs from progenitor cells during pregnancy.

#### Haemosphere

Haemosphere (https://www.haemosphere.org/) is an online web portal for the Haemopedia [24] and Haemopedia RNA sequencing (RNA-seq) [25] databases. Haemopedia contains microarray gene expression profiles of 169 mouse samples with 54 hematopoietic cell populations covering all major hematopoietic lineages (erythrocytes, megakaryocytes, mast cells, basophils, eosinophils, neutrophils, macrophages, dendritic cells, B cells, T cells, and natural killer cells, as well as progenitors and stem cells). Haemopedia RNA-seq consists of an RNA-seq dataset of 129 mouse samples with 57 cell populations spanning all major mature lineages and an RNA-seq dataset of 42 human samples with 12 cell populations covering 7 mature lineages (eosinophils, neutrophils, macrophages, dendritic cells, B cells, T cells, and natural killer cells). Moreover, Haemosphere has incorporated several publicly available hematopoietic RNA-seq datasets encompassing a range of normal hematopoietic cells, which can be used as reference datasets. Haemosphere provides a user-friendly interface for non-bioinformatics researchers to perform data analysis. Users can: (1) explore target gene expression across various datasets, cell types, and hematopoietic lineages; (2) identify differentially expressed genes under diverse conditions; (3) identify cell type-specific or lineage-specific genes (*i.e.*, highly expressed genes); and (4) compare gene correlations between two selected genes across different lineages.

#### Blood Proteoform Atlas

The Blood Proteoform Atlas (BPA, https://blood-proteoform-atlas.org/) [26] is a database of 568,113 redundant (29,620 non-redundant) proteoforms derived from 1690 genes across 21 distinct human hematopoietic cell types and plasma components. In the website, users can browse and search by gene, protein, proteoform, and cell type. And a presence/absence matrix of proteoforms across the cell types can be downloaded, allowing users to quickly screen for cell type-specific proteoforms. The BPA presents a reference map of proteoforms in human hematopoiesis, which makes it possible for researchers to get better biomarkers for diseases and treatments. This resource holds certain clinical application potential, such as tracking specific cells and protein forms involved in rejection reactions after organ transplantation.

#### StemDriver

StemDriver (http://biomedbdc.wchscu.cn/StemDriver/) [27] integrates 42 single-cell RNA-seq (scRNA-seq) datasets from humans and mice across 22 major cell types, encompassing the entirety of hematopoiesis and generates a knowledgebase of gene functions for HSC fate determination, including 23,839 human genes and 29,533 mouse genes. The function analysis includes (1) identification of highly expressed genes in specific cell types, (2) identification of genes with effective roles in cell differentiation, (3) calculation of gene importance across trajectories, and (4) identification of highly variable genes along the pseudotime. The results can be accessed and downloaded by clicking on the corresponding function block on the “Home” page of the StemDriver website. The analysis results of each dataset can be accessed through the “Dataset” page. On the “DriverMap” page, users can select specific cell types of interest to get more detailed information such as cell subtypes recorded, highly expressed genes, and associated datasets. A summary of the functional annotation for a gene can be accessed via the “Dataset” page or the “Gene” page.

### Resources for hematologic malignancies

#### Hemap

Hemap (http://hemap.uta.fi/) [28] is an interactive online resource for characterizing molecular phenotypes across hematologic malignancies. It integrates 7279 transcriptomes from 36 hematologic malignancies (leukemia, lymphoma, myeloma, and proliferative disorders), 862 from cancer cell lines, and 1403 from normal blood cell types, and faithfully groups these samples. The main analysis feature of Hemap is the pairwise exploration of cancer cluster, drug, and genomic pathway associations with various feature types to characterize the sample groups. The analysis results can be accessed by customizing cluster (“PW Cluster/Class” field) and feature (“Type” field) on the “Explore” interface, and individual feature can be selected directly for e-staining. The interface also supports e-staining by gene, pathway, and drug marker signatures, and allows users to overview gene sets of pathway and drug signatures. On the “Annotations” page, it allows users to flexible searches across all sample annotations, and the samples in the results can be added to the “GEXP boxplots” interface for gene expression visualization. For example, users can find subtype-specific druggable genes or cell population-specific genes.

#### ABC portal

ABC portal (http://abc.sklehabc.com) [29] is an important part of the Atlas of Blood Cells (ABC) project and is the first specialized single-cell database focused on blood and immune cells in human and mouse, which contains 111 normal hematopoiesis datasets and 87 disease-related datasets, and involves 12 blood diseases. Cell types of all datasets were re-annotated, including malignant cells. Annotation information can be downloaded via the button next to the dataset on the “Search” page of the website. In addition, ABC portal provides four interactive analysis modules on the detail page of each dataset: (1) Uniform Manifold Approximation and Projection (UMAP) module visualizes multi-level cell annotation and gene expression; (2) Composition module shows the cellular composition in each sample or each patient; (3) Signature expression module displays the scaled expression of genes in the selected signature gene set, such as the blood disease risk genes, Kyoto Encyclopedia of Genes and Genomes (KEGG) pathways, Gene Ontology (GO) terms, hallmark genes, and Reactome pathways; (4) Ligand–receptor (LR) network module exhibits the predicted LR network of selected cell types, including LR network, LR pair, and LR gene expression. Under the “Compare” page, users can select multiple datasets to compare cell composition and gene expression across samples. It is an important feature of ABC portal that allows reanalysis of user-selected subsets of data within a dataset or comparisons across datasets. At the end of the “Document” page, users can download cluster of differentiation (CD) marker genes for different cell types in human and mouse.

#### RNA Editome in Hematopoietic differentiation and malignancy database

RNA Editome in Hematopoietic differentiation and malignancy (REDH, http://www.redhdatabase.com) [30] database compiles 30,796 RNA editing sites of 4861 genes among 12 murine adult hematopoietic cell populations and 435,866 aberrant editing sites of 9126 genes associated with leukemia, including 10 diagnosed acute myeloid leukemia (AML) patients, 10 relapsed AML patients, 9 chronic myeloid leukemia (CML) patients, and 19 healthy donors. The detailed information of RNA editing sites as well as visualization of editing frequency and corresponding gene expression can be viewed and customized in the “Differentiation” and “Disease” modules, including queries for differentiation stage-specific and leukemia-specific differentially expressed editing sites. In the “Knowledgebase” module, it also organizes experimentally validated RNA editing events curated from multiple publications. Further, it provides functional enrichment results for RNA editing genes in the “Enrichment” module. Users can click on the enrichment result of interest to get the corresponding RNA editing site information.

### Leukemia-specific data resources

#### BloodSpot 3.0

BloodSpot [31–33] is a database of gene expression profiles for healthy hematopoiesis and leukemia. The newest version (BloodSpot 3.0, https://www.fobinf.com/) [34] expanded single-cell proteomics data as well as the AML and acute erythrocytic leukemia (AEL) datasets, including pediatric leukemia data. The web-based interface provides three concomitant levels of visualization for a gene query: (1) gene expression in different datasets or different cell populations; (2) survival analysis based on gene expression in TCGA-AML; and (3) an interactive hierarchical tree plot illustrating the relationship between displayed samples. Additionally, the interface displays the top correlating genes for the selected gene within the dataset via clicking on the “Gene Correlations” button.

#### BloodChIP Xtra

BloodChIP Xtra (https://bloodchipxtra.vafaeelab.com/) [35], the expanded version of BloodChIP [36] database, records and integrates chromatin immunoprecipitation sequencing (ChIP-seq) datasets profiling 10 transcription factors (TFs) and 3 histone modifications, assay for transposase-accessible chromatin with high throughput sequencing (ATAC-seq) datasets, and RNA-seq datasets — all were generated from rare primary human HSCs (*i.e.*, multipotent progenitor cells), hematopoietic progenitors (including common myeloid, granulocyte–macrophage, and megakaryocyte–erythrocyte progenitors), and 4 AML cell lines. On the “SEARCH” page of the web interface, the “Gene Database” box displays a summary table of 18,328 gene entries, including the number of annotated peaks (Binding Profiles), the University of California, Santa Cruz Genome Browser Database (UCSC) link for visualizing binding profiles (UCSC), gene expression, and summary of binding events (Binding Summary). Each gene entry can be expanded to show the genome coordinates of each peak and the binding status in each cell type. In the “Display Filters” check box, users can customize the binding profiles and cell types displayed. In addition, users can construct a more complex query by selecting genes or TFs in specific cell types. The retrieved TF binding information and normalized gene expression values can be exported for downstream analysis. The full data tables are available on the “DOWNLOAD” page.

#### LeukemiaDB

LeukemiaDB (http://bioinfo.life.hust.edu.cn/LeukemiaDB) [37] collects and integrates 188 leukemia-associated RNA-seq datasets covering 14 leukemia subtypes, 53 leukemia cell lines, and a total of 3068 samples (including 92 normal samples). It is the most comprehensive landscape of data resources for human leukemia to date, providing the expression profiles of protein-coding genes, long non-coding RNAs (lncRNAs), and circular RNAs (circRNAs), as well as the gene fusions and alternative splicing events for human leukemia transcriptomes. And the corresponding modules are provided in the website to visualize the expression level of all samples, average expression of all samples in patients and cell lines. When browsing by cell type or cell line, users can view the regulatory network (TF/lncRNA/circRNA–target) through “Regulatory modules” on the “Results” page. In addition, LeukemiaDB integrates TCGA-AML survival information and drug sensitivity data from Genomics of Drug Sensitivity in Cancer (GDSC) [38] and Cancer Therapeutics Response Portal (CTRP) [39], allowing users to understand the effects of selected genes on the survival of AML patients and the relationship to drug response in AML. The clear search functionality on the website is extremely user-friendly. For example, through the “Browse by leukemia subtypes” feature, users can directly view the specific and common characteristics exhibited by different leukemia subtypes.

### Comprehensive clinical resources

#### HemOnc.org

HemOnc.org (https://hemonc.org/wiki/Main_Page) [40] is a free, publicly available, comprehensive repository of chemotherapy information in the field of hematology/oncology, mainly including chemotherapy drugs and chemotherapy regimens. This information mainly is curated from published primary literature of prospective clinical trials in hematology/oncology, followed by some reviews and guidelines. In terms of drugs, it prioritizes the inclusion of approved anti-tumor drugs [US Food and Drug Administration (FDA) outperforms other regulatory agencies], followed by drugs with supportive effects in hematology/oncology, especially those used to address the side effects of anti-tumor drugs, and then those with good results in clinical trials. The “Drug details” page includes attributes, commonly used diseases, FDA-approved indications, and the generic names and brand names of the drug. For regimens, the publicly available and reliable systemic anti-cancer treatment regimens, as well as regimens that have undergone randomized clinical trials and have certain therapeutic effects, were embedded. On the “Disease search” page, all regimens were classified according to their evaluation environment, and each regimen lists specific medication information and clinical trial evidence.

#### European LeukemiaNet

The European LeukemiaNet (ELN, https://www.leukemia-net.org/) is an international collaborative network platform established to promote management of leukemia, partnering with approximately 220 institutions and over 10,000 doctors and researchers in leukemia research [41]. To accelerate the validation and dissemination of treatment progress, ELN collects data on basic research, clinical studies, and diagnostic methods in a unified and standardized manner. It has developed comprehensive guidelines and management recommendations for nearly all types of leukemia and interdisciplinary specialties. Additionally, ELN has created a clinical trial platform to integrate the performance of clinical trials of new drugs or treatment strategies across Europe. In addition, ELN regularly hosts international meetings, particularly the annual ELN symposium at no cost for participants.

#### My Cancer Genome

The My Cancer Genome (MCG, https://www.mycancergenome.org/) [42] is an online resource on precision cancer medicine knowledge for physicians, patients, caregivers, and researchers, and the first public resource to provide available, detailed information on cancer-related genetic variants for clinicians. MCG provides multiple interfaces for users to query, including “Clinical Trials”, “Diseases”, “Biomarkers”, “Drugs”, and “Pathways”. Currently, MCG encompasses data on 957 disease types, including 220 hematopoietic and lymphatic system neoplasms, 3324 biomarkers, 9852 clinical trials, 2876 drugs, and 19 biological pathways. On “Biomarkers” page, 3324 curated biomarkers were categorized into genes, genetic biomarkers, protein expression markers, chromosomal markers, and markers of genomic instability, which are very user-friendly. On the “Diseases” and “Drugs” pages, there is a “Biomarker-Directed Therapies” section that highlights disease-related biomarkers of susceptibility and resistance to therapeutic drugs. In addition, the “Drugs” page shows drug details and the clinical trials involved. The key cancer-related pathways affected by drugs can be found on the “Pathways” page. On the “Clinical Trials” page, users can quickly search for relevant clinical trials by selecting diseases, biomarkers, and drugs. Notably, the inclusion of clinical trials and cancer-relevant cell signaling pathways is a highlight of MCG.

## Blood cell image datasets

The use of image datasets is emerging as a key enabler in hematology and related disease research, especially in the areas of automatic classification, disease diagnosis, and cell type identification. In recent years, a variety of publicly available blood image datasets have provided a solid foundation for research in computer vision and deep learning techniques. Here, we review the characteristics of existing publicly available blood cell datasets for cell detection, segmentation, and classification, as detailed in Table 2.

## ML in hematology

### Integration of ML with blood cell images

#### ALNett

ALNett [54] is a customized convolutional neural network (CNN) model designed for the classification of acute lymphoblastic leukemia (ALL) using microscopic cell images. The model introduces a multilevel convolutional layer to enhance image feature extraction by capturing key patterns of cell morphology and distribution, and then uses a deep CNN to learn hierarchical features of the image. The model creates a multi-objective fitness function to balance multiple diagnostic metrics such as sensitivity and specificity. Compared to traditional ML methods and generalized CNNs, the classification accuracy and robustness of ALNett are superior with 95% accuracy and 93% F1 score. However, the performance of ALNett is influenced by the quality and diversity of training data, and it may encounter challenges with imbalanced datasets or noisy samples.

#### CMLcGAN

CMLcGAN [55] is a conditional generative adversarial network (cGAN)-based model designed to segment megakaryocytes from myeloid cells, enabling the diagnosis of CML. The model framework consists of a UNet++ generator to segment the cells and a multilayer CNN discriminator to classify the generator outputs. The model achieves an accuracy of 94.8% and an F1 score of 93.6%, outperforming traditional CNN-based methods, especially with limited data. However, there are challenges of labeling data dependency and generalization ability.

#### Leukocyte Feature-Extraction model

Leukocyte Feature-Extraction model (LeuFeatx) [56] is a Visual Geometry Group 16 (VGG16)-adapted fine-tuned feature-extractor model specifically designed to extract leukocyte-specific features. The fine-tuning process used the AML morphological dataset that comprises a diverse group of 15 cell types. Rastogi et al. [56] trained multiple classifiers using LeuFeatx deep features on three different datasets to evaluate the classification performance of the extracted features and compared the performance metrics of training each classifier with features from other extraction models. The results demonstrate that using LeuFeatx as a feature extractor achieves superior accuracy and specificity in both leukocyte subtype classification and binary classification tasks. However, the model’s performance is influenced by the quality and diversity of training data, and its reliance on high-performance computational resources may limit deployment in resource-constrained environments.

#### RedTell

RedTell (https://github.com/marrlab/redtell) [57] is an innovative artificial intelligence (AI) tool designed for interpretable analysis of red blood cell (RBC) morphology. It automates the segmentation and interpretable feature extraction of RBCs from microscopic images, and RBC classification. Sadafi et al. [57] demonstrated the applicability and capabilities of RedTell through three case studies, including extracting cellular features for different RBC diseases, classifying RBC subtypes, and distinguishing sickle cells. RedTell has shown excellent performance on several datasets, achieving an accuracy rate of 85%–92%, with high precision and recall to effectively reduce misdiagnosis. In addition, RedTell possesses interpretability, reflected in its segmentation results, feature extraction, and classification models, providing clear image overlays, handcrafted morphological features, and feature importance ranked by significance. However, this tool faces limitations in handling low-quality images and rare diseases.

#### Virtual hematological morphologist

Virtual hematological morphologist (VHM) is an AI-aided hematological diagnostic framework that integrates deep learning technologies with medical expertise [58]. The framework first utilizes a Faster Region-based CNN (Faster-RCNN)-based feature extraction model to analyze cellular morphological features in images, and then employs a support vector machine (SVM)-based case recognition model, integrating diagnostic criteria and extracted features, to achieve disease diagnosis. In multi-center datasets, it achieved over 95% classification accuracy, with a recall rate of 98% for common malignancies. The framework’s limitations include reduced performance in classifying rare cases and a high demand for computational resources.

### Integration of ML with omics data

Omics data, such as genomics, proteomics, and metabolomics, provide a wealth of potential information for understanding blood diseases. ML helps uncover the molecular mechanisms of diseases, predict disease progression, and discover new biomarkers by mining complex biological patterns from large-scale omics data. Mosquera Orgueira et al. [59] provided personalized survival prediction for AML patients using gene expression profiling. The random forest-based model identifies key gene expression features related to prognosis, enabling personalized survival prediction and supporting treatment decisions. Shreve et al. [60] developed a prognostic model for AML patients by combining clinical, gene expression, and mutation data, using XGBoost. Qin et al. [61] utilized bulk RNA-seq and scRNA-seq data, and clinicopathological information from AML patients to assess 10 ML models with 73 different algorithms, ultimately identifying 6 pan-programmed cell death-related genes (PPCDS) and developing a PPCDS index (PPCDI) model to predict the prognosis of AML. The results show that PPCDS is significantly correlated with malignant status of proliferative cells and a high PPCDI is associated with poor prognosis. Additionally, PPCDI can predict immunotherapy and targeted therapy response in AML.

### Integration of ML with clinical data

By combining clinical data with ML, we can improve disease prediction and treatment planning. This integration enables personalized healthcare, helping doctors make better decisions based on individual patient information. Warnat-Herresthal et al. [62] explored AML prediction by combining high-dimensional ML and a vast amount of gene expression data extracted from 12,029 samples. The results show excellent performance in identifying AML patients, with improved accuracy and robustness as data volume increases. Hauser et al. [63] used retrospective electronic health record (EHR) data and ML models (such as SVM and random forest) to predict CML. Through feature selection and deep learning frameworks, the model accurately identifies key features related to CML, supporting early diagnosis and personalized treatment. Shanbehzadeh et al. [64] compared various ML algorithms (such as SVM, random forest, and neural networks) for predicting 5-year survival in CML patients using clinical data, including patient demographics, treatment history, and laboratory results.

## Concluding remarks

While significant advancements have been made in integrating biological data resources with ML frameworks in hematology research, several critical limitations remain that hinder the full potential of these approaches. First, the heterogeneity in data quality and format — ranging from blood cell image annotations to the resolution and consistency of omics data — continues to pose challenges. This variability can lead to difficulties in training robust ML models and limit their generalizability across diverse patient populations and conditions. Furthermore, the integration of multi-modal data (*e.g.*, combining imaging, omics, and clinical data) often requires complex computational frameworks that are not yet fully optimized for clinical use, leading to slower adoption in real-world settings.

Another key limitation is the interpretability of ML models, which remains a significant barrier in clinical applications. Although deep learning algorithms can achieve high predictive accuracy, the lack of transparency in how these models arrive at their predictions makes it difficult for clinicians to trust and effectively use them in practice. Addressing these challenges requires a more concerted effort to develop explainable AI techniques that can offer insights into model decision-making processes while maintaining high accuracy.

Looking forward, future research should focus on addressing these gaps by improving data standardization and sharing practices to facilitate the creation of more diverse, high-quality datasets. Additionally, the development of advanced multi-modal deep learning models that can seamlessly integrate various types of biological and clinical data is crucial. Research efforts should also prioritize improving model transparency and explainability, ensuring that ML tools can be safely and confidently used in clinical settings. Finally, there is a need for real-time, actionable diagnostic tools that integrate ML models into clinical workflows, providing clinicians with timely insights for personalized treatment planning.

In summary, while the integration of ML with biological and clinical data has revolutionized hematology research, significant challenges remain. By focusing on data quality, model interpretability, and real-world applicability, future research can overcome these barriers and drive further progress in precision medicine for hematologic disorders. These advancements will not only enhance our understanding of hematologic diseases but also improve patient outcomes by facilitating more personalized and timely interventions.

## CRediT author statement

**Ying Yi:** Conceptualization, Data curation, Writing – original draft. **Yongfei Hu:** Writing – review & editing. **Juanjuan Kang:** Funding acquisition, Writing – review & editing. **Qifa Liu:** Funding acquisition, Writing – review & editing. **Yan Huang:** Writing – review & editing. **Dong Wang:** Supervision, Funding acquisition, Writing – review & editing. All authors have read and approved the final manuscript.

## Competing interests

The authors have declared no competing interests.
